# Nutrients as novel therapeutic approaches for metabolic disturbances in polycystic ovary syndrome

**DOI:** 10.17179/excli2016-422

**Published:** 2016-09-05

**Authors:** Asieh Mansour, Saeed Hosseini, Bagher Larijani, Mohammad Reza Mohajeri-Tehrani

**Affiliations:** 1 Department of Clinical Nutrition and Dietetics, Faculty of Nutrition and Food Technology, National Nutrition and Food Technology, Research Institute Shahid Beheshti University of Medical Science, Tehran, Iran; 2 Endocrinology and Metabolism Research Center, Endocrinology and Metabolism Clinical Sciences Institute, Tehran University of Medical Sciences, Tehran, Iran

**Keywords:** polycystic ovary syndrome, inositol, isoflavonids, resveratrol, vitamin D, PUFA

## Abstract

Polycystic ovary syndrome (PCOS) is one of the most common endocrine disorders among women. This disease is characterized by infertility, menstrual dysfunction, and hyperandrogenism. Also, PCOS is often associated with hyperlipidemia and impaired glucose tolerance, conditions that are associated with cardiovascular disorder, type 2 diabetes, cancer and hypertension. Evidence supports that some nutrients may affect the hormonal and metabolic disturbances of PCOS. Here in this study, we aimed to review the available literature that assessed the nutrients such as inostol, isoflavonids, resveratrol, vitamin D, and PUFA (polyunsaturated fatty acids), known to influence the hormonal and metabolic disturbances of PCOS, along with the strategies and future directions of nutrient supplementations in such patients.

## Abbreviations

BMI: Body mass index; DHA: Docosahexaenoic acid; DHEAS: Dehydroepiandrostrone sulfate; DNA: Deoxyribonucleic acid; EPA: Eicosapentaenoic acid; FAI: Free androgen index; FSH: Follicle stimulating hormone; GPx: Glutathione peroxides; GSH: Glutathione; HbA1c: Glycosylated hemoglobin; IGT: Impaired glucose tolerance; IPG: Inositolphosphoglycans; LDL-C: Low-density lipoprotein cholesterol; LH: Luteinizing hormone; OGTT: Oral glucose tolerance test; PCOS: Polycystic ovary syndrome; ROS: Reactive oxygen species; SOD: Superoxide dismutase; StAR: Steroidogenic acute regulatory; PPAR: Peroxisome proliferator-activated receptor; PRL: Prolactin; PUFA: Polyunsaturated fatty acids; SHBG: Sex hormone binding protein; T-l: Theca- interstitial; VEGF: Vascular endothelial growth factor 

## Introduction

Polycystic ovary syndrome (PCOS) is an endocrine disorder among women of reproductive age, affecting 6-10 % of women of reproductive age (Sabuncu et al., 2001[65]). This disease is clinically characterized by hyperandrogenic nature, chronic lack of ovulation and/or polycystic ovaries (Baillargeon et al., 2006[3]; Heimark et al., 2013[31]). This syndrome is frequently associated with metabolic disturbances such as insulin resistance and hyperinsulinemia (Kotsa et al., 2009[36]). The potentiality for developing impaired glucose tolerance or type 2 diabetes mellitus, before reaching the age of 26 years, exists in 40 % of obese PCOS patients (Diamanti-Kandarakis et al., 2006[15]). Symptoms of insulin resistance such as elevated blood pressure, obesity, and abdominal obesity are all the known risk factors for the development of metabolic syndrome, cardiovascular disorders and diabetes (Agarwal et al., 2012[1]). The exact mechanism(s) of this syndrome is obscure. However, insulin resistance is considered to be the main cause in the pathogenesis of this syndrome (Fenkci et al., 2003[21]). It has been postulated that elevated insulin (hyperinsulinemia) is important in the pathogenesis of endocrine abnormality in PCOS. Lowering insulin levels may produce favorable results in these patients (Vargas et al., 2011[77]). Despite the fact that there are no specific treatments available for this population, treatments are generally aimed at decreasing insulin and androgen levels. This encompasses drug therapy including insulin lowering and anti androgen medications or oral contraceptives, and life style interventions (Douglas et al., 2006[17]).

## Nutrients and PCOS

The effects of nutrient composition of diet on PCOS have started to receive attention only recently (Rodrigues et al., 2009[63]). Despite the evidence supporting an effect of nutrition therapy for patients with PCOS (O'Connor et al., 2010[50]), little is known about the influence of diet composition on PCOS metabolic and endocrine control (Rodrigues et al., 2009[63]). For individuals with PCOS, the dietary treatments plan focuses on macronutrients content. However, the focus on micronutrients also continues to be an effective strategy (Bernier, 2012[5]). Obviously, it is necessary to improve our understanding about functional roles of some specific nutrients in PCOS (O'Connor et al., 2010[50]). These aspects will be specially discussed in this paper in order to provide a comprehensive review of the recently published *in vitro* and *in vivo* animal studies and also human clinical trials in which the influence of novel nutrients such as inositol, isoflavonids, resveratrol, vitamin D, and PUFA (polyunsaturated fatty acids) on hormonal and metabolic disturbances of PCOS is assessed. 

### Inositol 

Studies using inositol are summarized in Table 1(Tab. 1) (References in Table 1: Costantino et al., 2009[13]; Genazzani et al., 2014[27]; Genazzani et al., 2008[25]; Nestler et al., 1999[48]; Genazzani et al., 2014[26]; Laganà et al., 2015[39]; Nordio and Proietti, 2012[49]; Pizzo et al., 2014[60]).

Two isomers of inositol (myo-inositol and D-chiro-inositol) are mediators of insulin action (Bizzarri and Carlomagno, 2014[7]). Myo-inositol is a nutrient belonging to vitamin B complex (Papaleo et al., 2009[58]). Myo-inositol is found in various types of foods (e.g. whole grains, seeds, and fruits) and also can be produced from glucose in the human body (Carlomagno and Unfer, 2011[10]). Evidence have shown that this nutrient could have a helpful role in decreasing the hormonal profile, oxidative abnormalities, and as well as the metabolic factors in patients with PCOS, probably due to the amelioration of insulin resistance in these patients (Costantino et al., 2009[13]; Donà et al., 2012[16]). In a placebo-controlled trial, women with PCOS were randomized to receive either oral myo-inositol (4 g/d) plus folic acid (400 mcg/d) or folic acid alone for 12-16 weeks (Costantino et al., 2009[13]). Results showed that myo-inositol administration diminished the serum androgen levels. Moreover, this treatment improved the glucose tolerance and other metabolic profiles of these women (Costantino et al., 2009[13]). Similar results were reported when the effects of myo-inositol (3 g/d) on hormonal profiles and insulin response during an oral glucose tolerance test (OGTT) in normal weight PCOS patients were analyzed (Genazzani et al., 2014[27]). Genazzani et al. (2008[25]) reported that myo-inositol supplementation (2 g/d) plus folic acid (200 µg/ d) was effective in the amelioration of plasma luteinizing hormone (LH), Prolactin (PRL), and testosterone levels in overweight PCOS patient after 12 weeks consumption, probably through the reduction in plasma insulin concentration (Genazzani et al., 2008[25]). The mechanism by which the myo-inositol induces its effect is probably through the induction of inositolphosphoglycans (IPG) release (Genazzani et al., 2008[25]). In fact, an IPG molecule containing D-chiro-inositol plays a key role in activating enzymes that control glucose uptake and usage. IPG performs as a putative post-receptor mediator of insulin signaling pathway or as a second-messenger (Baillargeon et al., 2010[4]; Papaleo et al., 2009[58]). Consequently, authors suggested that the insulin resistance observed in PCOS women is related, at least partially, to the defect in one of the mediators of insulin containing D-chiro-inositol (Bromberg and Edlich, 1994[8]). Metformin ameliorates insulin action in PCOS patients through releasing D-chiro-inositol-IPG mediator (Galazis et al., 2011[24]). Myo-inositol is the most common form of inositol, which is converted to D-chiro-inositol by an epimerase (Larner, 2002[40]). For the first time Bizzarri and Carlomagno (2014[7]) showed that D-chiro-inositol is reduced in the urine and tissues of non-insulin-dependent diabetic patients. A review examined the status of D-chiro-inositol in animals and suggested that the oral supplementation of D-chiro-inositol may perform to bypass an absence in conversion of myo-inoistol to d- chiro inositol. D-chiro-inositol administration may be effective in improving insulin resistance (Larner, 2002[40]). In the study of Nestler et al. (1999[48]), D-chiro-inositol taken orally (1200 mg/d) for 6-8 weeks, reduced the serum androgen levels and improved the insulin resistance associated with metabolic imbalances in obese women with the PCOS. Similar effects were observed with the consumption of small amounts of D-chiro-inositol (500 mg/d) for 12 weeks in obese hyperinsulinemia PCOS women (Genazzani et al., 2014[26]). Furthermore, in agreement with these findings, a very recent data by Laganà et al. (2015[39]) showed improved results in hormonal, metabolic, endocrine and the indices of ovarian function in PCOS women, following the oral ingestion of 1 gr of D-chiro-inositol plus 400 mcg of folic acid for 6 months. Surprisingly, a study performed in 50 overweight women with PCOS reported that 550 mg of myo-inositol plus 13.8 mg D-chiro-inositol in soft gel capsule consumed twice a day could better restore the metabolic parameters compared to the consumption of 2 g of myo-inositol in powder format. This observation indicates that the combination of such compound in physiological blood ratio (40:1) could be a useful tool for nutrition therapy of PCOS overweight patients, due to the beneficial effects of these agents on alleviation of metabolic syndrome risk (Nordio and Proietti, 2012[49]). Furthermore, the available data support the idea that both the inositol isoforms are effective in the treatment of patients with PCOS. Nevertheless, myo-inositol potentially improves the metabolic profile, whereas D-chiro-inositol exerts positive effects on hyperandrogenism (Pizzo et al., 2014[60]). Yet, a systematic review assessing the effects of D-chiro-inositol on ovulation and/or metabolic markers in PCOS failed to find consistent conclusion, mostly because of heterogeneity in the method of each study and also lack of relevant trials and small sample sizes (Galazis et al., 2011[24]). Finally, Unfer et al. (2012[76]) performed a meta-analysis of 6 randomized placebo-controlled trials which used a range of 0.2-4 g/day myo-inositol in PCOS patients and concluded that myo-inositol supplementation can decrease the levels of various hormones such as LH, LH/follicle stimulating hormone (FSH), PRL, and testosterone and improve the dyslipidemia by reducing insulin concentrations. Furthermore, authors suggested that 4 g/day myo-inositol treatment is more effective in the treatment of entire symptom spectrum. Interestingly, no side effects have been reported by doses used in all these studies (Carlomagno and Unfer, 2011[10]). 

Although, only few randomized controlled trials (RCTs) using small sample size have been conducted (Galazis et al., 2011[24]; Unfer et al., 2012[76]). Several studies have reported to date the positive effects of myo-inositol and D-chiro-inositol on clinical, metabolic, endocrine, hormonal, and oxidative abnormalities in women with PCOS. Based on these observations, this naturally occurring compound may represent an alternative or complementary care to metformin treatment in PCOS patients (Donà et al., 2012[16]).

### Isoflavonoids

Studies using isoflavonoids are summarized in Table 2(Tab. 2) (References in Table 2: Khani et al., 2011[35]; Kamel, 2013[33]; Romualdi et al., 2008[64]; Forouhari et al., 2013[23]).

Phytoestrogens are plant derived substances and include lignins, isoflavones and coumestans (Bhathena and Velasquez, 2002[6]). The isoflavonoids such as genistein and daidzein, which are mostly found in legumes such as soy beans and chickpeas, have received considerable attention in recent 2 decades (Eden, 2012[19]). A number of studies have shown improvement in insulin resistance and/or glycemic control in response to isoflavonegenistein consumption, a response that appears to be, at least partially, due to the positive effects of this agent on β-cells function through the promotion of proliferation and the inhibition of apoptosis in such cells (Gilbert and Liu, 2013[29]). Hence, phytoestrogens rich extracts may represent a promising candidate for the alternative or complementary management of conditions that are related to metabolic syndrome (Jungbauer and Medjakovic, 2014[32]). A quasi- randomized trial preformed in 146 subjects with PCOS revealed that 18 mg of genistein (twice a day) for 3 months compared with cellulose as a placebo could significantly decrease the serum concentrations of low-density lipoprotein cholesterol (LDL-C). Also, according to these results, LH, dehydroepiandrostrone sulfate (DHEAS), testosterone, and triglyceride levels were lower in patients after genistein consumption comparing with before the supplementation indicating that genistein could be a useful tool for nutrition therapy of POCS patients due to the beneficial effects on reproductive hormonal levels and also the improvement of lipid profiles (Khani et al., 2011[35]). These data are in line with the observation of Kamel (2013[33]) who found significantly favorable changes in LH level and FSH/LH ratio after ten days treatment with phytoestrogen in women with PCOS. In contrast, in the study of Romualdi et al. (2008[64]) 36 mg/d of genistein did not alter the hormonal milieu and glycoinsulinemic metabolism in PCOS subjects. The authors concluded that the major influence of genistien seems to be on blood cholesterol. Then, a controlled 2 months cross-over trial conducted by Forouhari et al. (2013[23]) failed to show the effect of diet rich in isoflavones (70 g/d soy flour) on FSH, estradiol, and testosterone concentrations in women with PCOS as compared to control group. 

In conclusion, these data provide evidences that isoflavonegenistein could represent a therapeutic strategy for the treatment of clinical and metabolic imbalances in PCOS patients. The beneficial effect was associated with the improvement of lipid profile, although it was not necessarily occurred in hormonal milieu. Long term trials are needed to evaluate the effects of isoflavone on hormonal, oxidative, and metabolic abnormalities in PCOS women.

### Resveratrol

Resveratrol (trans-3,5,4'-trihydroxystilbene) is a naturally occurring phytoalexin that is produced by some plants such as grapes, nuts and berries in response to injury or fungal infection (Palsamy and Subramanian, 2010[57]; Svechnikov et al., 2009[69]). Resveratrol is thought to have a number of incredible health benefits, including: antioxidant, anti-inflammatory, anti-cancer, anti-aging and cardio-protection (Oskarsson et al., 2014[55]; Palsamy and Subramanian, 2010[57]; Svechnikov et al., 2009[69]).

Wong et al. (2010[79]) found that resveratrol increased the apoptosis of ovarian theca-interstitial (T-l) cells and prevented the cell proliferation *in vitro* at concentration 30-100 µM. The antiproliferative actions of resveratrol might be more pronounced among PCOS patients, whose ovarian function is disturbed as a result of excessive T-l cells production (Wong et al., 2010[79]). In contrast with this data, Ortega et al. (2012[54]) observed minimal effects of resveratrol on the apoptosis of rat granulosa cells. The authors suggested that the discrepancy observed among their results obtained on granulosa cells, and the previous study which used theca-interstitial cells could be due to the different effects of resveratrol on different type of cells. Additionally, resveratrol showed that *in vitro* it could inhibit the secretion of estrogen and vascular endothelial growth factor (VEGF) in rat granulose cells (Ortega et al., 2012[54]). Thence, biological effects of resveratrol on ovarian cells that may result in changes of the balance between relative ratios of such cells and decrease of VEGF expression can have pervasive consequences in PCOS condition (Ortega et al., 2012[54]). 

There is a broad range of experimental evidences for resveratrol as steroid hormones inhibitor *in vitro* (Ortega et al., 2012[54]; Oskarsson et al., 2014[55]; Svechnikov et al., 2009[69]) with several different mechanisms; at least in part, through the reduction of Cyp19 mRNA expression (Ortega et al., 2012[54]), and as another mechanism, suppression of StAR (steroidogenic acute regulatory) protein and cytochrome P450c17 expression (Svechnikov et al., 2009[69]). Molecular mechanism(s) of action of resveratrol may be different, depending on resveratrol concentration (Schmitt and Dirsch, 2009[67]) and/or the type of cell (Liu et al., 2013[42]). Ortega et al. (2014[53]) indicated that the combination of resveratrol and simvastatin is a highly effective androstenedione and androsterone production inhibitor in rat theca-interstitial cells compared to simvastatin alone. A study investigating the role of resveratrol in the PCOS was conducted on PCOS models (Ergenoglu et al., 2015[20]). The administration of resveratrol lowered serum superoxide dismutase (SOD) activity and elevated glutathione peroxides (GPx) level. Moreover, a reduction in the levels of plasma anti-Mullerian hormone and insulin-like growth factor 1, besides in the number of antral follicle was observed in PCOS rats. The authors concluded that due to its antioxidant properties resveratrol supplementation had therapeutic effect on experimental PCOS induced by dihydrotestosterone (Ergenoglu et al., 2015[20]). 

There is evidence that resveratrol exerts suppressive action on insulin release in both *in vitro *and* in vivo* (Szkudelski, 2006[70], 2008[71]). The insulin suppressive effect is beneficial for patients who suffer from exaggerated secretion of insulin such as patients who have insulinoma or PCOS (Szkudelski, 2008[71]). In disagreement with this finding, the oral administration of resveratrol (5 mg/kg body weight) for 30 days in Wistar diabetic rats resulted in the enhancement of insulin secretion and antioxidant competence in islet β cells compared to control rats (Palsamy and Subramanian, 2010[57]). A very recent meta-analysis of clinical trials showed significant advantages of resveratrol in the improvement of glycosylated hemoglobin (HbA1c) (Hausenblas, et al., 2014[30]). Since nearly 30-40 percent of PCOS patients have impaired glucose tolerance (IGT) and 7.5-10 percent of them are found to have type 2 diabetes (Sirmans and Pate, 2014[68]), the benefits associated with the consumption of resveratrol for PCOS patients with type 2 diabetes gets more clear.

Collectively, this data provide evidences that resveratrol supplementation could represent a novel treatment for the management of PCOS patients mostly because of its antioxidant properties (Wong et al., 2010[79]). Indeed, to the best of our knowledge, there has been no published trial, so far, examining the influence of resveratrol on the condition associated with PCOS outcome. However, two studies are registered with clinical trials registry to assess the effects of resveratrol on biochemical factors, reproductive as well as endocrine outcomes in PCOS population (Ortega and Duleba, 2015[52]). Thus, future research is needed to assess the resveratrol effects on PCOS symptoms. 

### Vitamin D

Studies using vitamin D are summarized in Table 3(Tab. 3) (References in Table 3: Kotsa et al., 2009[36]; Rashidi et al., 2009[62]; Firouzabadi et al., 2012[22]; Ardabili et al., 2012[2]; Raja-Khan et al., 2014[61]).

Vitamin D deficiency (25OHD<20 ng/ ml) is very common in women with PCOS so that 67-85 % of women with PCOS have low levels of vitamin D (Mahmoudi et al., 2010[44]; Thomson et al., 2012[73]). Vitamin D insufficiency could contribute to the development of insulin resistance and obesity (Tzotzas et al., 2010[75]; Wehr et al., 2009[78]), as the major features of PCOS (Wehr et al., 2009[78]). Besides, it has been reported that gene polymorphism is linked with PCOS (at least partially) through the role of this gene on insulin blood levels and insulin resistance (Mahmoudi, 2009[45]). Yet, the mechanism(s) by which low vitamin D levels can cause insulin resistance is unclear (Lerchbaum and Obermayer-Pietsch, 2012[41]). In an animal study, it has been suggested that vitamin D could pose as an effective antioxidant by enhancing the levels of glutathione (GSH), SOD, and GPx and also by diminishing in lipid peroxidation, and notably, the antioxidant effect of vitamin D was much greater than that the effect observed for vitamin E (Sardar et al., 1995[66]). In human study, Tarcin et al. (2009[72]) suggested that vitamin D could act as a potent antioxidant through the inhibition of oxidative stress and lipid peroxidation. Given that chronic inflammation and oxidative stress play causative roles in pathogenesis of insulin resistance (Ceriello and Motz, 2004[11]) and on the other hand, vitamin D has effects on insulin levels and expression of insulin receptor, and also inhibits inflammation (Tzotzas et al., 2010[75]) and oxidative stress (Lerchbaum and Obermayer-Pietsch, 2012[41]). For the first time, Kotsa et al. (2009[36]) showed that the consumption of Alphacalcidol (1-a-hydroxyvitamin D3) 1 µg/day for 3 months in 15 obese women with PCOS and insulin resistance, produced an effect on the first phase insulin stimulation, indicating that vitamin D3 could be effective in the treatment of PCOS. Rashidi et al. (2009[62]) determined that combined consumption of metformin 1500 mg/d plus calcium 1000 mg/d and vitamin D 400IU/d was more effective in the treatment of PCOS, as indicated by the maturation of follicles, than either compound alone. Similarly, Firouzabadi et al. (2012[22]) reported that in 100 infertile PCOS women, following the intake of metformin 1500 mg/d plus calcium 1000 mg/d and vitamin D 100000 IU/month for 6 months, body mass index (BMI) was significantly reduced and follicle maturation and hyperandrogenism was improved in comparison with those treated with metformin 1500 mg/d alone. Therefore, abnormal calcium homeostasis is associated with clinical manifestations of PCOS including follicular arrest and the menstruation disorders (Thys-Jacobs et al., 1999[74]). Serum 25-hydroxy vitamin D level is lower in obese PCOS women (Yildizhan et al., 2009[80]) and associates inversely with insulin resistance (Tzotzas et al., 2010[75]). On the other hand, moderate weight loss can increase 25 hydroxy vitamin D levels and this raise is correlated with beneficial effects on insulin sensitivity (Tzotzas et al., 2010[75]). Hence, life style interventions such as weight management program (Tzotzas et al., 2010[75]) and vitamin D administration can be useful to treat obese PCOS patients (Yildizhan et al., 2009[80]) and PCOS women with low 25(OH)D serum levels (Brzozowska and Karowicz-Bilińska, 2013[9]). Finally, a very recent systematic review examined the effects of vitamin D on metabolic abnormalities and suggested an inverse association between these two parameters in women suffering from PCOS (Krul-Poel et al., 2013[37]). Yet, some studies failed to support these results; Ardabili et al. (2012[2]) demonstrated that low dose vitamin D (50000 IU/every 20 day) did not improve insulin resistance after 2 months supplementation in women with PCOS and vitamin D deficiency. Raja-Khan et al. (2014[61]) reported that high dose vitamin D (12000 IU/d) supplementation did not improve insulin sensitivity in PCOS patients. Taken together, further well designed clinical studies are needed to find out the effect of vitamin D supplementation in treating PCOS patients (Krul-Poel et al., 2013[37]; Thomson et al., 2012[73]).

### PUFA

Studies using PUFA are summarized in Table 4(Tab. 4) (References in Table 4: Kasim-Karakas et al., 2004[34]; Mohammadi et al., 2012[46]; Nadjarzadeh et al., 2013[47]; Oner and Muderris, 2013[51]; Cussons et al., 2009[14]; Kuzmanov, 2009[38]; Vargas et al., 2011[77]).

Dietary intake of PUFA, counting n-3 and n-6 fatty acids, was positively correlated with GPx activity (Chen et al., 2003[12]). It seems that PUFA is involved in the suppression of apoptosis which is responsible for pathophysiology of PCOS (Ghasemzadeh et al., 2013[28]). Moreover, it is interesting to note that PUFAs improve the action of insulin in peripheral target organs and reduce insulin secretion from ß-cells of the pancreatic. In addition, PUFA and their products such as 15-dexyprostaglydin J_2_ seem to act as natural ligands for Peroxisome Proliferator-Activated Receptor (PPAR) gamma. Due to the treatment application of synthetic PPAR gamma ligand used in insulin resistance therapy in PCOS population, it can be assumed that dietary PUFAs may play a role in the reduction of insulin resistance (Kasim-Karakas et al., 2004[34]). However, one research evaluated the effects of PUFA rich diet (48 g walnuts/800 kcal of total calorie intake) in 17 PCOS patients after a 3 months habitual diet. No change in plasma testosterone nor LH and FSH levels was detected after 6 months (Kasim-Karakas et al., 2004[34]). Higher circulating n-6 and higher ratio of n-6 to n-3 are shown to be associated with higher plasma androgen levels in PCOS patients, and testosterone plasma levels reduced by n-3 PUFA administration (Phelan et al., 2011[59]). Omega 3 PUFA can be considered as an important PUFA involved in regulating insulin production and action, as well as improving inflammatory processes (Vargas et al., 2011[77]). Oral supplementation of n-3 PUFA plus lower carbohydrate feeding has been shown to improve PCOS in rats; with a decrease in levels of testosterone and increase in levels of FSH (Ouladsahebmadarek et al., 2014[56]). In a study by Mohammadi et al. (2012[46]) it was shown that supplementation with long chain omega-3 fatty acid (EPA and DHA) had beneficial effects on cardiovascular risk through the improvement of antioxidant levels, insulin resistance and lipid profile without changing the body weight in women with PCOS. In another study with 78 overweight and obese PCOS patients, omega n-3 fatty acid (3 g/day) consumption decreased testosterone concentration compared with placebo, while no significant changes in free androgen index (FAI) and sex hormone binding protein (SHBG) levels occurred in either treatments (Nadjarzadeh et al., 2013[47]). Likewise, in a study with PCOS women, the results supported the efficacy of omega-3 supplementation (1500 mg/day) in reducing insulin and HOMA levels and improving hormonal profile after 6 months of treatment (Oner and Muderris, 2013[51]). In a study by Cussons et al. (2009[14]) supplementation at higher doses (omega-3 PUFA 4 g/d) in 25 women with PCOS reduced levels of triglysecride and hepatic fat. Moreover, a recent review concluded that omega-3 seems to improve dyslipidemia and insulin sensitivity in PCOS patients by producing anti-inflammatory and antioxidant activity (Macut et al., 2012[43]). 

However, the results of Kuzmanov study (2009[38]) showed that the consumption of omega-3 fatty acids did not have a significant impact on the reproductive or metabolic abnormalities of PCOS after 3 months of therapy. 6 week of treatment with a daily long chain omega-3 PUFA applying a dose of 3.5 g in PCOS population didn't affect insulin levels and fasting glucose (Vargas et al., 2011[77]). For the first time, Dunaif et al. (1995[18]) suggested that excessive insulin receptor serine phosphorylation is responsible for defects in insulin action, and also shows that serine phosorylation of IRS-1 is actually increased in women with PCOS. It is tempting to speculate that PCOS individuals couldn't reverse this abnormality, and omega-3 PUFA couldn't accordingly improve insulin resistance (Vargas et al., 2011[77]). However, it remains a need to conduct a comparative study to evaluate the contradictory effects of n-3 PUFA on PCOS and healthy control women (Vargas et al., 2011[77]).

Although it appears that omega-3 fatty acids may improve metabolic profiles and hormonal outcomes in PCOS patients, not all studies have supported the efficiency of omega-3 PUFA. For example, its effect on insulin action is vague. Prior to be recommended, more research needs to be explored in this specific field. 

## Conclusion

Since oxidative stress, metabolic, hormonal and endocrine imbalance has been implicated in the development of PCOS, antioxidant agents and nutrients that improve such abnormality have the potential to reduce the risk of this syndrome. Findings of different several studies suggest that the nutrients are not equally effective in improving hormonal and metabolic disturbances of PCOS. As an example, among the nutrient studies in PCOS, inositol has shown the most promise. Yet, PUFA administration had been less effective to improve hormonal imbalances in such patients. The combined effects of specific nutrients in PCOS women need to be investigated in future studies. Finally, additional researches using antioxidants interventions such as vitamin E, vitamin C, and lycopene are warranted in PCOS patients. 

## Conflict of interest

The authors declare no conflict of interests.

## Figures and Tables

**Table 1:**
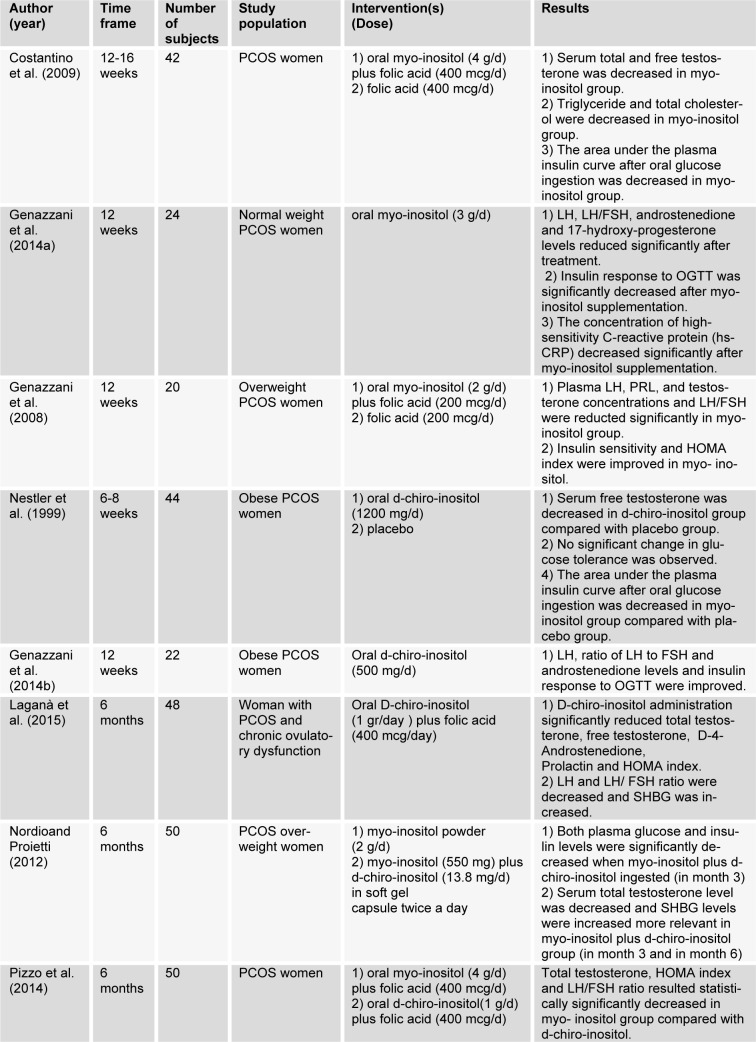
Metabolic and hormonal effects of inositol in PCOS

**Table 2:**
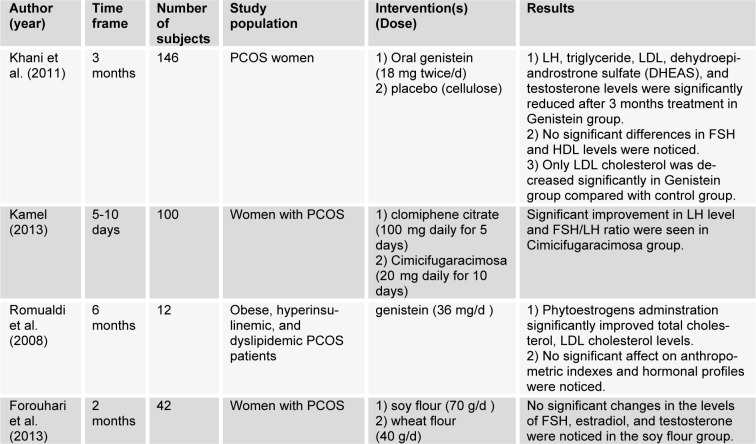
Metabolic and hormonal effects of isoflavonoids in PCOS

**Table 3:**
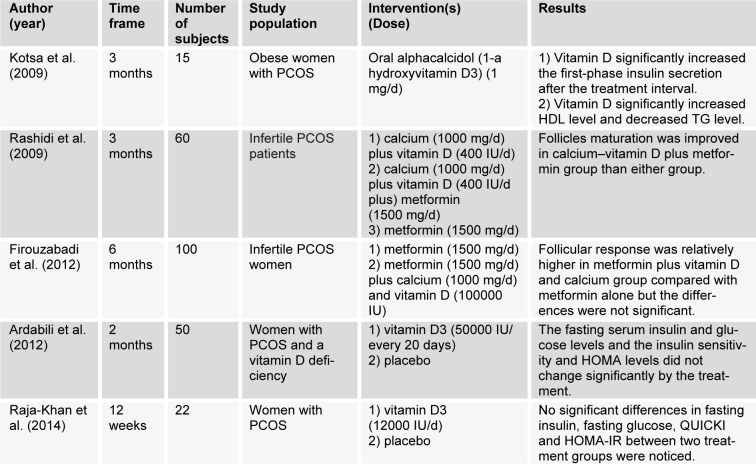
Metabolic and hormonal effects of vitamin D in PCOS

**Table 4:**
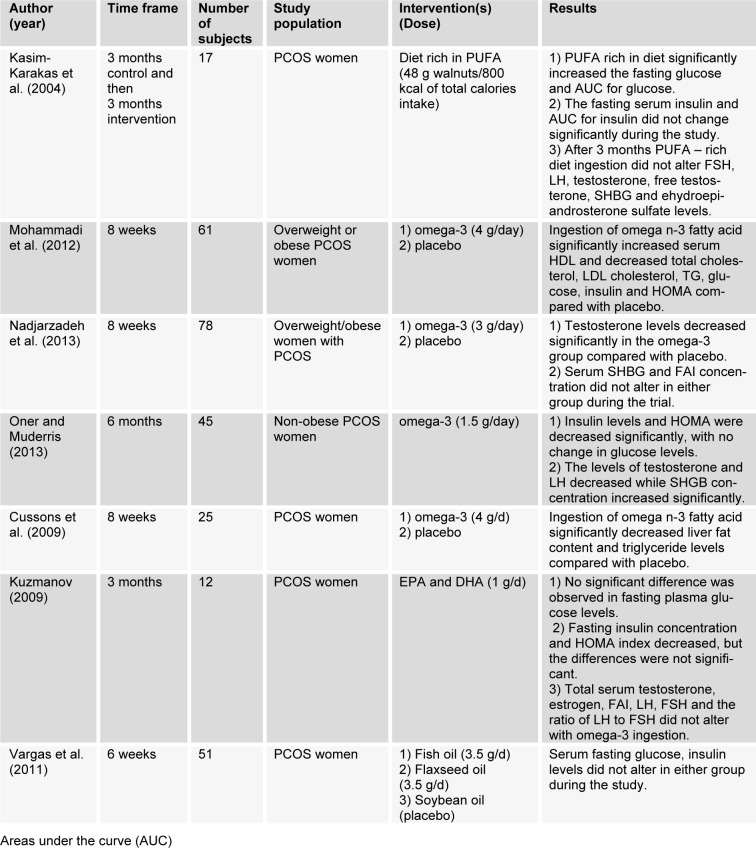
Metabolic and hormonal effects of PUFA in PCOS
